# Dengue serotype-specific immune response in *Aedes aegypti* and *Aedes albopictus*

**DOI:** 10.1590/0074-02760170182

**Published:** 2017-12

**Authors:** Chelsea T Smartt, Dongyoung Shin, Barry W Alto

**Affiliations:** University of Florida, Department of Entomology and Nematology, Florida Medical Entomology Laboratory, Vero Beach, FL, USA

**Keywords:** dengue, serotype, Aedes aegypti, Aedes albopictus, antiviral response

## Abstract

**BACKGROUND:**

Dengue viruses (DENV) are considered one of the most important emerging pathogens and dengue disease is a global health threat. The geographic expansion of dengue viruses has led to co-circulation of all four dengue serotypes making it imperative that new DENV control strategies be devised.

**OBJECTIVES:**

Here we characterize dengue serotype-specific innate immune responses in *Aedes aegypti* and *Aedes albopictus* using DENV from Puerto Rico (PR).

**METHODS:**

*Ae. aegypti* and *Ae. albopictus* were infected with dengue serotype 1 and 2 isolated from Puerto Rico. DENV infected mosquito samples were collected and temporal change in expression of selected innate immune response pathway genes analyzed by quantitative real time PCR.

**FINDINGS:**

The Toll pathway is involved in anti-dengue response in *Ae. aegypti,* and *Ae. albopictus.* Infections with PR DENV- 1 elicited a stronger response from genes of the Toll immune pathway than PR DENV-2 in *Ae. aegypti* but in infected *Ae. albopictus* expression of Toll pathway genes tended to be similar between the serotypes. Two genes (a ribosomal S5 protein gene and a nimrod-like gene) from *Ae. albopictus* were expressed in response to DENV.

**MAIN CONCLUSIONS:**

These studies revealed a role for antiviral genes in DENV serotype-specific interactions with DENV vectors, demonstrated that infections with DENV-2 can modulate the Toll immune response pathway in *Ae. aegypti* and elucidated candidate molecules that might be used to interfere with serotype specific vector-virus interactions.

Dengue viruses (DENV, genus *Flavivirus*, family *Flaviviridae*) are considered one of the most important emerging pathogens (WHO 2016). There are four antigenically distinct serotypes of DENV (DENV-1 - 4), which are further divided into distinct genotypes within each serotype (Rico-Hesse 2003). Dengue viruses are maintained in a transmission cycle that generally includes the primary mosquito vectors, *Aedes aegypti* and to a lesser extent *Ae. albopictus,* and humans who serve as amplification hosts (Christofferson 2015). Transmission of DENV is endemic throughout tropical and subtropical regions and results in 390 million infections per year (Bhatt et al. 2013, WHO 2016). Dengue disease is found in over 100 countries of which regions including the Americas, South-east Asia and Western Pacific are the most seriously affected. Recently there has been an increase in the number of dengue cases, with over 2.35 million reported in the Americas in 2013. Additionally, dengue has spread to new regions such as Europe, with local transmission reported in France and Croatia in 2010 and in 2012 a dengue outbreak was reported in Portugal that resulted in over 2000 cases (WHO 2016).

Overlap in the spatial distribution of antigenically distinct serotypes of DENV (1 - 4) have been implicated in changes in disease incidence, severity and infectiousness (Thu et al. 2004, Barr et al. 2012). A change in serotype prevalence from one outbreak to another in Myanmar was also shown to impact severity of disease and incidence. Thu et al. (2004) found that during one of the largest DENV outbreaks in Myanmar, 95% of the DENV isolated from patients with clinical disease were serotype 1 belonging to two genetic lineages, representing a higher number than in outbreaks of the previous four years when all of the dengue serotypes were detected (Thu et al. 2004). In addition, there were no patients with DENV–4, there were more dengue hemorrhagic fever (DHF) cases compared to dengue shock syndrome cases, and a larger proportion of patients had primary DENV infections. Nucleotide differences between DENV-1 from the Myanmar outbreak and DENV-1 from other Myanmar localities revealed 46 amino acid changes. Of these, eight amino acid changes distinguished the two lineages responsible for the 2001 outbreak with all other Myanmar DENV-1. Taken together this suggests that a rapid genetic change in the DENV-1 genotype occurred resulting in the appearance of a more virulent and pathogenic virus. A DENV outbreak in Puerto Rico in 2010 also revealed serotype-specific disease severity and the highest incidence of reported cases and deaths in this region (Sharp et al. 2013). The epidemic in 2010 was mainly attributed to primary infections with DENV-1 and predominantly found in 1-4-year-old children. Dengue positive cases detected by reverse transcription polymerase chain reaction (RT-PCR) revealed that most blood samples from infected patients contained DENV-1 (69%) followed by DENV-4 (23%), and DENV-2 (7%) where each serotype exhibited some degree of regional specificity to incidence (Sharp et al. 2013). There were few DENV–3 positive samples (less than 0.1%) in the 2010 outbreak. DENV-4 infected more patients with DHF and resulted in more severe illness than DENV-1, with individuals aged 10-19 years old being most affected. Sequence analysis revealed that the 2010 isolates of DENV-1 and −4 belong to clades that were distinct from those serotypes isolated during the 1998 Puerto Rico epidemic.

Vector competence of a mosquito for DENV is also influenced by serotype and genotype (Moncayo et al. 2004, de Castro et al. 2013, Whitehorn et al. 2015, Vazeille et al. 2016). Whitehorn et al. (2015) orally exposed *Ae. aegypti* and *Ae. albopictus* mosquitoes to DENV by allowing them to feed on viremic humans known to be infected with the different serotypes of DENV. The results showed that *Ae. albopictus* was less likely to have abdominal infections and infectious saliva with DENV–2 and −4 than *Ae. aegypti,* while both were equally likely to develop infectious saliva from DENV-1 and −3 (Whitehorn et al. 2015). Genotype-specific differences in DENV virulence is hypothesized to have allowed for competitive displacement of existing DENV strains by more virulent DENV strains associated with more severe dengue disease in humans. For example, the displacement of American DENV-2 genotype by Southeast Asian DENV-2 genotype is associated with higher virus replication, higher susceptibility to infection and dissemination, and a shorter extrinsic incubation period in *Ae. aegypti* (Armstrong & Rico-Hesse 2003, Anderson & Rico-Hesse 2006). Similarly, in Sri Lanka a native DENV-3 strain was displaced by an invasive DENV-3 strain. Both DENV strains were associated with similar rates of infection in *Ae. aegypti,* but the invasive DENV-3 strain had a greater replicative advantage and induced greater disseminated infections (Hanley et al. 2008).

A comparison of endemic versus sylvatic DENV-2 strains was performed as a retrospective test of the hypothesis that dengue emergence is mediated by adaptation to peridomestic mosquitoes (Moncayo et al. 2004). This study showed higher susceptibility to infection in both *Ae. aegypti* and *Ae. albopictus* with endemic than sylvatic DENV-2. Collectively, these studies provide compelling evidence to support the notion that there may be huge variation in vector competence related to DENV serotype and genotype (Vazeille et al. 2016).

Ramírez and Dimopolous (2010) revealed that the Toll innate immune pathway responded to different serotypes of DENV, and that Toll pathway factors act as anti-DENV molecules in laboratory and field-derived *Ae. aegypti.* However, the influence of serotype was performed in a lab derived mosquito colony and the Toll response between the populations of mosquitoes included only one serotype.

Few studies show the interaction of multiple serotypes isolated from one geographic region on a specific population of mosquitoes when looking at the influence of different serotypes on infection in mosquitoes and few have compared antiviral responses between two closely related DENV-competent species. Recently such a study was performed using two distinct DENV serotypes from Puerto Rico and *Ae. aegypti* and *Ae. albopictus* (Alto et al. 2014). The results showed no significant differences in infection or dissemination rates between *Ae. aegypti* and *Ae. albopictus* infected with DENV-1. *Ae. aegypti* were significantly more susceptible to midgut infection with DENV-2 and had a higher dissemination rate than *Ae. albopictus*, suggesting that *Ae. albopictus* is a less efficient potential transmitter of DENV-2 from Puerto Rico (Alto et al. 2014).

Here we extend this study to investigate the underlying molecular mechanisms responsible for the variation in the mosquito -DENV serotype interaction via molecular characterization of genes involved in known antiviral responses in DENV infected *Ae. aegypti* and *Ae. albopictus.*

## MATERIALS AND METHODS

*Mosquitoes* - Larval *Ae. aegypti* and *Ae. albopictus* were collected from water-filled containers in Key West (Old Town historic district) and Vero Beach, FL, respectively. Mosquitoes were reared using methods described by Alto et al. (2014). Briefly, mosquitoes were kept at 26-28°C and 60-80% relative humidity in a climate controlled room and a light:dark cycle of 14:10 hours. Larvae were reared in enamel pans with ca. one liter tap water and 0.4 g larval food consisting of equal amounts of brewer's yeast and lactalbumin and supplemented with the same amount 3-4 days later. Approximately 150 larvae were reared together in pans. Adults were maintained in 0.3m^3^ cages with 20% sucrose solution from cotton wicks and weekly blood meals from chickens (IACUC protocol 201507682) to propagate eggs used to initiate the experiments.

*Infection of Ae. aegypti and Ae. albopictus* - Five to six day-old female *Ae. aegypti* (F2) and *Ae. albopictus* (F4) in four treatment groups (100 females/treatment group) were fed defibrinated bovine blood (Hemostat, Dixon, CA) containing one of the two serotypes, DENV-1 and −2 (GenBank accession #s EU482591, EU482553) that were isolated from Puerto Rico (PR) and provided by the Centers for Disease Control and Prevention (PR) as described by Alto et al. (2014). Infectious blood meals were delivered to *Aedes* mosquitoes using an artificial feeding apparatus (Hemotek, Lancashire, United Kingdom) with hog casing membranes as previously described (Alto et al. 2014). Control blood meals were prepared similarly, but without the presence of DENV. After feeding, mosquitoes were cold anesthetized and fully engorged specimens were transferred to one-liter cardboard cages with mesh screening, maintained in incubators at 28°C, and provided a 20% sucrose solution for the duration of the experiment. A subset of the fully engorged mosquitoes were collected at 4, 8, and 18 h post infection (hpi) and 1, 3, 5, and 7 days post infection (dpi), and RNA extracted to determine susceptibility to infection, disseminated infection, viral titer in mosquito bodies, infection and dissemination rates and the targeted gene expression. The amount of DENV containing blood imbibed by mosquitoes was determined by destructively sampling freshly blood-fed mosquitoes and quantifying viral titer by quantitative RT-PCR standardized with plaque assay. Mosquitoes imbibed 6.8 ± 0.5 and 7.1 ± 1.2 log10 plaque forming unit equivalents (pfue) DENV/mL for PR DENV-1 and PR DENV-2, respectively (Alto et al. 2014). The DENV titers in the infected blood meals did not significantly differ between the two serotypes (p > 0.05). Infection rates of mosquito bodies (indicator of susceptibility to infection) and legs (indicator of viral disseminated infection beyond the midgut to other tissues and organs) collected at 7 and 14 dpi using DENV serotype-specific primers is presented in another study (Alto et al. 2014). DENV titration in samples collected at the earlier time points was performed using the iTaq^TM^ Universal SYBR Green One-Step Kit (BioRad, Hercules, CA) on the Bio-Rad CFX96^TM^ Real-Time PCR Detection System with DENV specific primers and following a standard protocol (Alto et al. 2014, Shin et al. 2014). The standard curves for DENV titer was obtained by serial dilution of DENV stock (6.8 ± 0.5 and 7.1 ± 1.2 log10 pfu for PR DENV-1 and PR DENV-2, respectively). The standard curves were defined as the regression line of the logarithm of standard copy number *versus* Ct value. The titers of infected bodies from *Ae. aegypti* and *Ae. albopictus* at each time point were determined with the standard curve generated from PR DENV-1 and PR DENV-2.

*RNA extraction and gene expression analysis* - Twelve individual DENV-infected female mosquitoes were collected at each time point/biological replicate (triplication, 36 total samples) for subsequent RNA extraction to determine the effect of DENV serotype on infection and innate immune response related gene expression. Individual female bodies were homogenized by hand using an Eppendorf tube mortar and pestle and RNA extracted using Trizol Reagent (Invitrogen, Carlsbad, CA) using previously established methods (Shin et al. 2014). Quantitative real time polymerase chain reaction (qRT-PCR) was performed on cDNA, synthesized with Enhanced Avian Reverse Transcriptase (Sigma, St. Louis, MO) and qRT-PCR conducted using SsoAdvanced SYBR Green Supermix (Bio-Rad, Hercules, CA) and primers specific to the antiviral genes of interest (Table I) (Nene et al. 2007) on the Bio-Rad CFX96^TM^ Real-Time PCR Detection System and following the included protocols. Two technical replicates were performed for all qRT-PCR reactions. The standard curve is generated based on the expression of the *Ae. aegypti* ribosomal protein S7 gene (GenBank Accession # AY380336). Primers to known antiviral genes were designed based on the *Ae. aegypti* genome as the *Ae. albopictus* genome is not fully annotated. Primer compatibility was verified using blast analysis to find highly similar genes in the *Ae. albopictus* database, and *vice versa*. Primer pairs designed to one species that proved unsuccessful in product amplification in the second species were redesigned to be species specific (Table II). Melting curves from qRT-PCR reactions were evaluated for redundant primer binding. The expression of each gene was compared with non-infectious (i.e., no DENV present) blood-fed mosquitoes. The time points post-infection were based on previous studies showing the temporal progression of infection in *Ae. aegypti* under these conditions (Richards et al. 2012, Alto et al. 2014).

**TABLE I:** Mosquito gene primer sets and sequences used in quantitative real-time polymerase chain reaction (qRT-PCR)

Primer name	Primer sequence
AEG hypo Sptz5 F	5'- TCC TTC GCC ATC TCC CTT CAA GTT −3'
AEG hypo Sptz5 R	5'- AGT TGC CCT TGG TGT TCA AAG CTG −3'
AEG cactus F	5'- AGA CAG CCG CAC CTT CGA TTC C −3'
AEG cactus R	5'- CGC TTC GGT AGC CTC GTG GAT C −3'
AEG Rel1A-F	5'- AGA AAG CCA TGT CCG ATC TGG TGA −3'
AEG Rel1A-R	5'- CCT GTT TGT GCA CGT TGG TAT GCT −3'
AEG dome F2	5'- GCA TCA GCG GGA AAG TTC CAA TGT −3'
AEG dome R2	5'- AGC TTG TAA TCG GTG GGA ATC GGA −3'
ribosomal S5 F (Set 4)	5'- CCG AAT GCT TGG CTG ATG A −3'
ribosomal S5 R (Set 4)	5'- GAC ACC TTA TCG GTT GGA CTT-3'
GR881776 F (Set 2)	5'- GAC TGG ATG TGA GCC GAT TT −3'
GR881776 R (Set 2)	5'- CAC GTA GCG TCA CAT TTG TTT C −3'
RPS7-F	5'-GGG ACA AAT CGG CCA GGC TAT C-3'
RPS7-R	5'-TCG TGG ACG CTT CTG CTT GTT G-3'
AAL dome FWD Set 2	5'-GTG CAA TAG TCG AAG GGT ACA G-3'
AAL dome REV Set 2	5'-GTA CGG CTT CAG TCC AGT TAT G-3'
AAL sptz5 FWD Set 3	5'-CCT AAA CAC CAA GGG CAA CT-3'
AAL sptz5 REV Set 3	5'-CTC GCT TGA CGT GCA TAT CT-3'
AAL cactus FWD Set 1	5'-GAC TCT AGC CAG CAT TCT TGT-3'
AAL cactus REV Set 1	5'-TGG GCT TCA GCT GCA TAT AG-3'
AEG AAEL007967 FWD Set 1	5'-CAG GTG ACA ATC GCT GTA GAT-3'
AEG AAEL007967 REV Set 1	5'-GTC CTA CAC ACT GCC CAT TTA-3'

**TABLE II:** Mosquito genes tested in the expression assays with dengue viruses-infected (DENV-infected) mosquitoes

Accession No.	Source	Gene name	Gene description
AAEL001929	*Aedes aegypti*	Spätzle	Toll receptor binding ligand
AALF008627	*Ae. albopictus*		
AAEL000709	*Ae. aegypti*	Cactus	Negative regulator of the Toll pathway in mosquitoes
AALF007886	*Ae. albopictus*		
AAEL007696	*Ae. aegypti*	Rel 1A	Intracellular NF-kB-like factor; % ID to *Ae. albopictus* AALF009555=77.3%
AAEL012471	*Ae. aegypti*	Domeless	Transmembrane receptor activating the Janus kinase pathway;
AALF011453	*Ae. albopictus*		
AF263471	*Ae. albopictus*	ribosomal S5 protein mRNA	Translation; % ID to *Ae. aegypti* AAEL013625=82%
GR881776	*Ae. albopictus*	putative innate	Unknown
AAEL007967	*Ae. aegypti*	immune related gene	

*Statistical analysis* - The gene expression in each group at each time point was compared by Mann- Whitney U test. Kruskal-Wallis and Dunn's multiple comparison tests, using the GraphPad Prism statistical software package (Prism 7.0; GraphPad Software, Inc.), were used to calculate p-values and determine the significance of the titers from each group, consisting of two serotypes, two *Aedes* species, and seven time-points.

*Ethics* - The animal protocols used in this work were evaluated and approved by the Institutional Animal Care and Use Committee (IACUC) at the University of Florida (IACUC Study #201507682). The treatment of animals in this study was in compliance with the Laboratory Animal Welfare Act of 1966 (P.L. 89-544).

## RESULTS

The influence of dengue serotype on antiviral responses between the two major vectors, *Ae. aegypti* and *Ae. albopictus,* was investigated (Xi et al. 2008, Souza-Neto et al. 2009). Pathogen infection response genes shown to have differential expression following infection of *Ae. aegypti* and *Ae. albopictus* (Xi et al. 2008, Souza-Neto et al. 2009, Dixit et al. 2011), including spätzle, cactus, relish, domeless, an *Ae. albopictus* ribosomal S5 protein gene, and nimrod-like gene, were investigated for changes in expression over seven time points during seven days following exposure to DENV serotypes 1 and 2 from PR (Table II). Our analyses did not include all members of the innate immune response pathways nor was the expression of all genes detected in both species under these conditions.

**Fig. 1:**
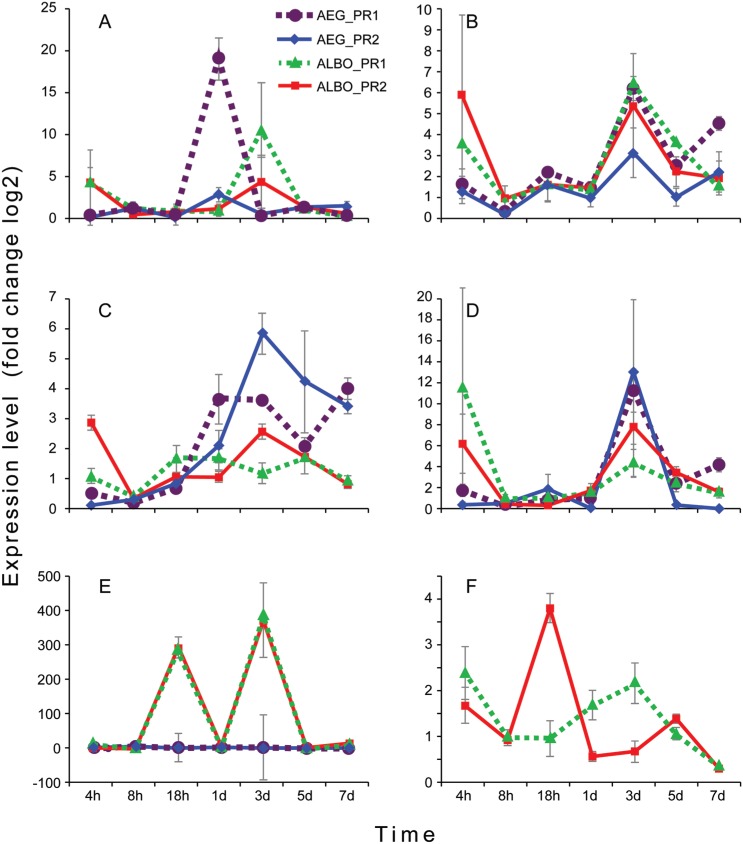
temporal fold change in expression of the mosquito innate immune response genes in female *Aedes aegypti* and *Ae. albopictus* following infection with either dengue viruses-1 (DENV-1) or DENV-2 from Puerto Rico. The colored lines represent the mosquito species-DENV serotype combinations used in the study: (A) spatzle expression; (B) cactus expression; (C) relish1A expression; (D) domeless expression; (E) *Ae. albopictus* ribosomal S5 protein gene expression; (F) *Ae. albopictus* putative innate immune related gene expression. AEG: *Ae. aegypti;* ALBO: *Ae. albopictus;* DENV-1: PR1; DENV-2: PR2; h: hour; d: day.

Expression of spätzle, a ligand that binds the Toll receptor in the Toll innate immune response pathway (Xi et al. 2008), was analyzed in *Ae. aegypti* mosquitoes infected with PR DEnV-1 and −2. The highest fold change in expression of this gene resulting from infection with either virus strain was detected at 1dpi (Fig. 1A) with PR DENV-1 having higher levels compared to PR DENV-2, although the level differences were not significant. Significant differences in spätzle expression between *Ae. aegypti* infected with PR DENV-1 and −2 was seen at 4 hpi (p < 0.05) (Supplementary data, Table). Expression of spätzle did not increase in *Ae. aegypti* infected with either virus after 1 dpi. The spätzle gene from *Ae. albopictus*, with high similarity to *Ae. aegypti* spätzle (89.6%)(VectorBase, Blastn) was assessed for expression changes in *Ae. albopictus* infected with the same virus as mentioned previously using species-specific primers. At 4 hpi, spätzle expression showed > 3 fold change in expression in *Ae. albopictus* infected with both DENV serotypes (p < 0.05). Highest fold change in expression was detected at 3 dpi for both DENV serotypes, although infection with PR DENV-1 had a significantly higher adjustment in expression (p < 0.05) (Fig. 1A, Supplementary data, Table). However, the difference in spätzle expression in DENV infected *Ae. albopictus* were only significant at 5 dpi with mosquitoes infected with PR DENV-2 having higher expression (p < 0.05) (Supplementary data, Table).

Expression of cactus, a negative regulator of the Toll pathway in mosquitoes (Xi et al. 2008), was investigated in the same DENV infected *Ae. aegypti* and *Ae. albopictus* mosquitoes. Cactus expression detected in *Ae. albopictus* mosquito samples determined using primers designed to the *Ae. albopictus* cactus gene was highest in expression change at 3 dpi in mosquitoes infected with both DENV serotypes, however > 10 fold change in expression was significant in samples infected with DENV-1 (p < 0.05) (Fig. 1B). Significant cactus expression differences in DENV infected *Ae. albopictus* were only present at 5 dpi. *Aedes aegypti* mosquitoes infected with PR DENV-1 had a similar pattern of cactus gene expression as those infected with PR DENV-2 but, at 3 and 7 dpi, expression was significantly higher compared to mosquitoes infected with PR DENV-2 (p < 0.05) (Fig. 1B, Supplementary data, Table). The highest fold change in cactus expression occurred at 3 dpi in *Ae. aegypti* infected with PR DENV-1 (p < 0.05).

Rel1A is an intracellular NF-kB-like factor involved in transcription of antimicrobial peptides in the nucleus as part of the Toll immune response pathway (Xi et al. 2008) and was shown to share 77.3% identity between the *Ae. aegypti* and *Ae. albopictus* gene (VectorBase, Blastn). Rel1A expression in *Ae. aegypti* mosquitoes infected with PR DENV-1 and −2 showed a down-regulated gene expression pattern but increased at 3 dpi (p < 0.05), however the differences in expression were only significant at 4 hpi and 8 hpi (Supplementary data, Table). *Ae. aegypti* mosquitoes infected with PR DENV-1 and PR DENV-2 showed highest Rel1A expression levels at 7 and 3 dpi, respectively (Fig. 1C). Additionally, Rel1A gene expression between *Ae. aegypti* mosquitoes infected with PR DENV-1 and PR DENV-2 was significantly different at 3 dpi (p < 0.05). When *Ae. albopictus* were infected and expression of Rel1A determined using primers designed to *Ae. aegypti* Re1A gene, PR DENV-1 did not elicit a change in expression above 2, while infection with PR DENV-2 elicited higher fold change in expression at 4 hpi and 3 dpi than infection with PR DENV-1 (p < 0.05) (Fig. 1C).

Domeless (Dome) is a transmembrane receptor that binds unpaired ligand (UPD) to activate the Janus kinase -signal transduction and activators of transcription (JAK-STAT) pathway against arboviruses (Souza-Neto et al. 2009). The Dome gene from *Ae. aegypti* mosquitoes shares 85% identity to Dome from *Ae. albopictus* mosquitoes (VectorBase, Blastn). *Ae. aegypti* mosquitoes infected with PR DENV-1 and DENV-2 showed highest gene expression level for Dome at 3 dpi (p < 0.05) (Fig. 1D) and there was no significant difference between the two serotypes. For samples infected with PR DENV-1, the change in Dome expression was significantly higher at 1, 5 and 7 dpi compared to mosquitoes infected with PR DENV-2 (p < 0.05) (Fig. 1D, Supplementary data, Table). Dome expression in *Ae. albopictus* mosquitoes infected with PR DENV-1 determined using primers designed to *Ae. albopictus Dome* showed highest fold change in expression at 4 hpi while mosquitoes infected with PR dEnV-2 showed highest fold change at 3 dpi (p < 0.05). Significant differences in Dome expression between *Ae. albopictus* infected with both DENV serotypes was seen at 8 hpi and 5 dpi (Supplementary data, Table). Regardless of mosquito species and serotype, greater than 4-fold change in expression of Dome was evident at 3 dpi (p < 0.05) and expression decreased through 7 dpi (Fig. 1D).

An *Ae. albopictus* ribosomal S5 protein gene was reported to be differentially expressed during DENV-2 infections (GeneBank Accession # AF263471). Vector-Base Blastn analysis revealed an 82% identity between *Ae. albopictus* ribosomal S5 protein gene used in this study and *Ae. aegypti* 40S ribosomal protein S5 gene. Analysis of the expression of the ribosomal S5 protein gene with primers designed to *Ae. albopictus* in the same samples infected with the DENV serotypes mentioned previously revealed high fold change in expression at 8 hpi for *Ae. aegypti* infected with PR DENV-1 and −2, while for *Ae. albopictus* the highest fold change in expression was shown at 3 dpi, with an increase in expression of more than 100 times at 18 hpi and 3 dpi compared with *Ae. aegypti* at the same time points (p < 0.05) (Fig. 1E). The expression level of the ribosomal S5 protein gene in *Ae. aegypti* and *Ae. albopictus* showed a similar trend within species without showing significant differences between DENV strains (Fig. 1E).

Expression of an *Ae. albopictus* gene suggested to be part of the innate immune response and whose expression is upregulated in C6/36 *Ae. albopictus* cells after bacterial exposure (GeneBank Accession # GR881776) (Dixit et al. 2011) was investigated in *Ae. aegypti* and *Ae. albopictus* mosquitoes infected with the DENV strains mentioned previously to verify if this gene has a role in general immune response or if it is bacterial specific. Blast analysis of the putative translation product of this gene revealed identity to the nimrod-gene family (60%, NCBI Blastx) of *Anopheles gambiae* (Estévez-Lao & Hillyer 2014) and to a gene of unknown function from *Ae. aegypti* (80%, Accession # AAEL007967, VectorBase Blastn). Expression of this gene, using primers specific to AAL007967, was not detected in *Ae. aegypti* mosquitoes infected with either DENV strain. Infection of *Ae. albopictus* with PR DENV-1 and PR DENV-2, exhibited a change in expression at 4 hpi that dramatically decreased by 8 hpi (Fig. 1F). Infection with PR DENV-1 revealed a gradual increase in expression of this gene until 3 dpi then expression decreased. PR DENV-2 infection in *Ae. albopictus* revealed the highest fold change in expression at 18 hpi (p < 0.05) that decreased rapidly by 1 dpi and remained low during the remaining time points (Fig. 1F). Significant differences in the expression of the *Ae. albopictus* nimrod-like gene between *Ae. albopictus* infected with the DENV serotypes was seen at 18 hpi, 1 and 3 dpi.

**Fig. 2:**
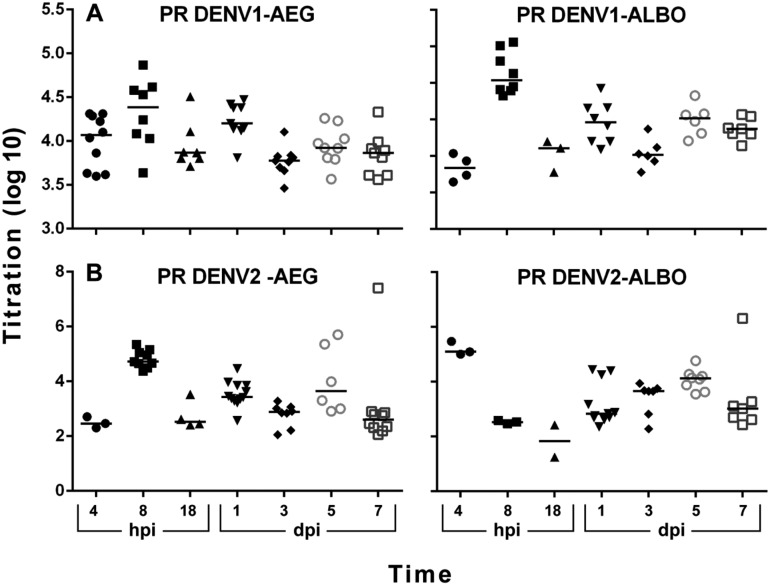
change in titration of dengue viruses-1 (DENV-1) (A) and DENV-2 (B) in individual *Aedes aegypti* (AEG) and *Ae. albopictus* (ALBO) mosquitoes over time. PR: Puerto Rico; hpi: hours post infection; dpi: days post infection. X axis: time; Y axis: Log_10_ pfue/mL of mosquito homogenate. The median of DENV titers is represented by a line. Each symbol represents an individual mosquito.

*Comparative DENV titration in Aedes species* - The titers of each dengue serotype in the two mosquito populations were evaluated in the same samples that were used in the gene expression studies (Fig. 2). Although the titration results from *Ae. aegypti* infected with PR DENV-1 showed few within-serotype differences over time, the means of the overall virus titers from infections with PR DENV-1 were significantly higher than PR DENV-2 (p < 0.05) (Fig. 2A-B). Titration of infection in *Ae. albopictus* tended to be similar within-serotype and infections with PR DENV-1 had higher titers, although they were not significant. However, the titer of PR DENV-2 in *Ae. albopictus* at 8 hpi (5.0 log10 plaque-forming unit (pfu) equivalents DENV/mL) was significantly lower than other groups at the same time point except for *Ae. aegypti* infected with PR DENV-1 (p < 0.05).

## DISCUSSION

Specific members of the innate immune response pathways shown to be involved in anti-dengue responses (Xi et al. 2008, Estévez-Lao & Hillyer 2014, Liu et al. 2015) were investigated in *Ae. aegypti* and *Ae. albopictus* mosquitoes to determine if the various immune responses to DENV differ in these two species and to determine if serotype affects their antiviral response. The expression of two *Ae. albopictus* genes shown previously to be expressed following DENV-2 and microbial challenge (Dixit et al. 2011) were also investigated. Under these conditions, we were not able to detect expression of the *Ae. albopictus* nimrod-like gene in *Ae. aegypti*. This could suggest that undetected genes have low expression limits or sequence differences.

Activation of spätzle to bind the Toll receptor initiates the antiviral cascade following DENV invasion (Xi et al. 2008). Infection of *Ae. aegypti* showed highest spätzle gene expression level at 1 dpi regardless of serotype (DENV-1 or −2) supporting its role in antiviral responses and implicating involvement in viral invasion and replication. Also, the different expression levels of this gene at 1 dpi between DENV-1 and −2 may be responsive to the infection rate between two serotype, because *Ae. aegypti* showed lower antiviral gene expression with DENV-2 infection but showed a higher infection rate (Alto et al. 2014). High spätzle expression in infected *Ae. albopictus* at 3 dpi suggests that the spätzle gene in *Ae. albopictus* responds to DENV virus however a role in antiviral response is only apparent for mosquitoes infected with PR DENV-1.

Expression of the negative regulator of the Toll pathway, cactus, was investigated in the two mosquito species under the same infection regime. Cactus expression in *Ae. aegypti* showed a similar pattern over time in response to each DENV serotype. However, the difference in expression level of this gene supports involvement of cactus in antiviral processes and indicates a serotype-specific response to infection. This gene is known as a negative regulator of Toll antiviral response but up-regulation of cactus gene expression (3-7 dpi) in DENV infected *Ae. aegypti* samples in this study may suggest an additional role in the mosquito infection process besides as a negative regulator as its expression decreased in response to viral invasion after 7 dpi (Fig. 2, Xi et al. 2008). Previous work showed that there were higher PR DENV-1 titers in bodies than PR DENV-2 (Alto et al. 2014) and suggests that PR DENV-2 might control the antiviral response in these *Ae. aegypti* ensuring that this species is successful in spreading DENV-2 (Ramírez & Dimopoulos 2010). The lower titer in bodies infected with PR DENV-2 might also result from expression of mosquito microRNA molecules that have been shown to regulate genes involved in virus replication and dissemination (Campbell et al. 2014). Cactus expression in *Ae. albopictus* was similar in response to each DENV serotype, with highest expression at 3 dpi coinciding with a negative change in titer for PR DENV-1 but positive change in PR DENV-2 titers (Fig. 2) suggesting a differential response due to serotype.

Rel1A is the initiator of antimicrobial peptide transcription used to combat a DENV infection. Therefore, upon DENV infection Rel1A expression is upregulated (Xi et al. 2008). Results from this study revealed upregulation of expression of Rel1A in response to PR DENV strain infection in both mosquito species after 1 dpi, although expression of Rel1A was differentially regulated between *Ae. aegypti* and *Ae. albopictus.* Additionally, DENV-2 elicited a higher fold change in expression than DENV-1, suggesting that Rel1A expression is controlled by viral load in *Ae. aegypti* but not for *Ae. albopictus* as this study showed higher body titers for infection with PR DENV-1 than PR DENV-2 in *Ae. aegypti* (Fig. 2, Alto et al. 2014).

Infection with two different serotypes resulted in changes in gene expression of the Toll immune pathway factors suggesting a serotype-specific Toll pathway response in the *Ae. aegypti* Key West population (Carvalho-Leandro et al. 2012, Whitehorn et al. 2015). Differential response by Toll pathway factors could indicate suppression of the pathway by the DENV-2 serotype or differences in growth kinetics between the two viral serotypes (Ramírez & Dimopoulos 2010, Shin et al. 2013, Campbell et al. 2014).

The JAK-STAT pathway is also involved in antiviral response in mosquitoes. Analysis of the expression of one component of this pathway, Dome (Lin et al. 2004, Souza-Neto et al. 2009), in DENV infected *Ae. aegypti* revealed few serotype specific expression differences, however there were significant differences at 3 time points. Dome expression in DENV infected *Ae. albopictus* was similar between serotypes, with only two time points having significant expression differences supporting a role in antiviral response that is not serotype specific. In the current study, *Ae. aegypti* and *Ae. albopictus* samples infected with both PR DENV serotypes showed upregulated expression of Dome at 3 dpi suggesting that activation of JAK-STAT pathway in response to infection with the dengue PR strain might be involved in dissemination (Salazar et al. 2007, Ramírez & Dimopoulos 2010). Surprisingly at 7 dpi Dome expression in *Ae. aegypti* mosquitoes infected with PR DENV-1 was upregulated while expression in *Ae. aegypti* mosquitoes infected with PR DENV-2 was severely decreased (Fig. 1D). Although the titers were not significantly different at this time point, the infection rate differences between serotype −1 and 2 supports this result (Alto et al. 2014). Thus, the differences in Dome expression could indicate that PR DENV-2 is better able to avoid antiviral responses than PR DENV-1 perhaps by controlling the immune response mechanism (Ramírez & Dimopoulos 2010, Carvalho-Leandro et al. 2012) and supports that *Ae. aegypti* is a better vector of DENV −2 than DENV-1 (Whitehorn et al. 2015).

Response to DENV infection by an *Ae. albopictus* ribosomal S5 protein gene, previously found to respond to DENV-2 infection in C6/36 cells was investigated in both mosquito species. This ribosomal protein gene has a translation function in mosquito cells and, due to current research on the use of ribosomal proteins in anti-viral therapeutics (Lee et al. 2013), is an interesting candidate for future research. Infection with PR DENV elicited a fold increase in expression in both species however for *Ae. aegypti* the change occurred during the early time points, regardless of viral serotype. The change early in the infection cycle in *Ae. aegypti* could indicate that the gene plays a role in movement of virus into the midgut epithelial cells or replication, as indicated by the change in DENV titer, but not dissemination as the expression decreased after 1 dpi (Fig. 1E, Fig. 2, Supplementary data, Table). Expression of the gene in infected *Ae. albopictus* elicited serotype specific expression at the first time point, as there was a 15 fold change in expression after infection with PR DENV-1 at 4 hpi compared to infections with PR DENV-2. However, expression after 8 hpi was similar regardless of DENV serotype. There were two notable increases in expression for the ribosomal S5 protein gene, at 18 hpi and 3 dpi, suggesting a role in DENV replication as there was a change in DENV titer at these time points (Fig. 2). Expression was increased again at 7 dpi, implicating a possible antiviral role during dissemination of DENV as the titer decreased at this time point for both serotypes. Based on titration data of the DENV serotypes at the same time point, the fold change in expression had little to do with change in overall titer (Fig. 2). The alteration in expression of this gene in both species following infection is support for its involvement in DENV-induced responses in these mosquitoes.

The expression of an *Ae. albopictus* gene shown to respond to bacterial exposure with identity to the nimrod gene family was investigated for its potential antiviral function. Infection of *Ae. aegypti* with the DENV serotypes used in this study did not initiate the expression of the *Ae. albopictus* nimrod-like gene suggesting no involvement in DENV infection in this mosquito species. However, analysis of the expression of the *Ae. albopictus* nimrod-like gene in DENV infected *Ae. albopictus* revealed serotype-specific differences in expression, with highest expression detected early in the infection process regardless of viral serotype. The upregulation of the *Ae. albopictus* nimrod-like gene expression early in the DENV infection cycle suggests response to the midgut infection and replication process (Salazar et al. 2007).

We have previously shown that both *Ae. aegypti* and *Ae. albopictus* are readily infected with PR DENV-1 and −2 however there were titer differences in mosquito samples at 7 dpi and 14 dpi (Alto et al. 2014), suggesting the presence of species-DENV strain interactions. Work in this paper corroborates this and reveals that the differences might be due to dissimilar antiviral pathway activation and response time, which we have shown to vary between species and by DENV serotype. In general, infection with two different serotypes elicited a stronger Toll pathway response in *Ae. aegypti* and this trend was also seen in similarly infected *Ae. albopictus* mosquitoes which suggests that PR DENV-2 has either a slower replication rate than PR DENV-1, a titer threshold, or is able to regulate the mosquito antiviral response. Also, this study supports involvement of the JAK-STAT pathway in antiviral responses in *Ae. aegypti* and *Ae. albopictus* infected with DENV. We have characterized the temporal expression of two *Ae. albopictus* genes in DENV infected mosquitoes, enabling, for the first time, their designation as antiviral factors in mosquitoes.
