# Supplementary material for: Dengue serotype-specific immune response in Aedes aegypti and Aedes albopictus
Source: Mem Inst Oswaldo Cruz. 2017 Dec;112(12):829–37. doi: 10.1590/0074-02760170182 (PMC5719552; doi:10.1590/0074-02760170182)
Supplement: Supplementary file 1 [file 0074-0276-mioc-112-12-0829-Suppl01.pdf]

SUPPLEMENTARY TABLE

Descriptive statistics for gene expression data from *Aedes aegypti* and *Ae. albopictus* infected with dengue virus-1 and 2 (DENV-1, 2)

	Average and standard deviation of each gene by species and serotypes at each time point								Mann-Whitney test results of the gene expression data		
	AEG _PR1		AEG_PR2		ALBO_PR1		ALBO_PR2			AEG	ALBO
	average	std	average	std	average	std	average	std		PR1 vs PR2	PR1 vs PR2
SPTZ									SPTZ		
4hpi	0,370	0,346	0,143	0,909	4,21476	0,944507	4,322499	1,938924	4hpi	0,0495	0,5127
8hpi	1,272	0,315	1,209	0,362	1,225624	0,391457	0,492941	0,103741	8hpi	0,8273	0,0707
18hpi	0,377	0,281	0,106	0,854	0,913509	0,4185	0,839944	0,331032	18hpi	0,2752	0,8273
1dpi	19,020	2,518	2,858	0,850	0,852129	0,263079	1,192911	0,277678	1dpi	0,0833	0,4404
3dpi	0,381	0,358	0,562	0,156	10,49494	2,849499	4,409869	1,579606	3dpi	0,2752	0,1011
5dpi	1,357	0,436	1,332	0,595	1,063673	0,03245	1,413951	0,111469	5dpi	0,8273	0,0045
7dpi	0,369	0,258	1,516	0,531	0,269121	0,10317	0,628674	0,138263	7dpi	0,5127	0,1011
Cactus									Cactus		
4hpi	1,661	0,708	1,304	0,588	3,59598	1,329715	5,862139	3,842751	4hpi	0,5127	0,5127
8hpi	0,331	0,050	0,201	0,082	0,865982	0,373099	0,951128	0,612083	8hpi	0,2752	0,8336
18hpi	2,219	0,114	1,619	0,769	1,59198	1,120653	1,628154	0,835205	18hpi	0,5127	0,8273
1dpi	1,453	0,261	0,969	0,406	1,352413	0,321822	1,464476	0,30113	1dpi	0,2752	0,4622
3dpi	6,204	0,570	3,139	1,184	6,428144	1,568962	5,395472	2,468855	3dpi	0,0495	0,3017
5dpi	2,513	0,429	1,021	0,428	3,616869	1,147752	2,238559	0,710707	5dpi	0,1260	0,0446
7dpi	4,538	0,326	2,226	0,961	1,460015	1,078963	1,938498	0,810226	7dpi	0,0495	0,5688
Rel1A									Rel1A		
4hpi	0,491	0,102	0,108	0,265	1,070	0,047	2,869	0,231	4hpi	0,0495	0,0495
8hpi	0,192	0,032	0,315	0,053	0,360	0,016	0,328	0,009	8hpi	0,0495	0,8273
18hpi	0,665	0,066	0,844	0,471	1,663	0,229	1,056	0,013	18hpi	0,5127	0,2752
1dpi	3,645	0,826	2,060	0,462	1,711	0,513	1,022	0,186	1dpi	0,1266	0,2752
3dpi	3,609	0,233	5,846	0,344	1,169	0,694	2,555	0,257	3dpi	0,0495	0,0495
5dpi	2,089	0,252	4,236	0,605	1,729	1,716	1,707	0,190	5dpi	0,2752	0,8273
7dpi	4,017	0,351	3,421	0,130	0,945	0,292	0,798	0,157	7dpi	0,2752	0,5127

	Average and standard deviation of each gene by species and serotypes at each time point								Mann-Whitney test results of the gene expression data		
	AEG _PR1		AEG_PR2		ALBO_PR1		ALBO_PR2			AEG	ALBO
	average	std	average	std	average	std	average	std		PR1 vs PR2	PR1 vs PR2
Dome									Dome		
4hpi	1,738	0,582	0,364	0,275	11,59398	9,452075	6,191066	2,82615	4hpi	0,1266	0,5127
8hpi	0,199	0,042	0,486	0,224	0,949032	0,391591	0,43181	0,221141	8hpi	0,5127	0,0272
18hpi	0,826	0,104	1,878	1,377	0,997217	0,415745	0,301135	0,314396	18hpi	0,5127	0,1266
1dpi	1,001	0,249	0,027	0,014	1,484586	0,316527	1,71981	0,674246	1dpi	0,0495	0,9578
3dpi	11,243	2,050	13,028	6,898	4,353354	1,288778	7,827005	4,844842	3dpi	0,8273	0,1797
5dpi	2,379	0,262	0,340	0,151	2,363398	0,758824	3,435787	0,572411	5dpi	0,0495	0,0321
7dpi	4,181	0,666	0,000	0,000	1,48694	0,466099	1,588813	0,358842	7dpi	0,0495	0,6547
Ribosomal S5									Ribosomal S5		
4hpi	2,578	0,842	2,215	0,110	15,775	0,723	0,465	0,060	4hpi	0,5127	0,5127
8hpi	5,323	0,502	5,858	0,264	0,941	0,158	0,796	0,110	8hpi	0,5127	0,8273
18hpi	0,989	0,028	0,933	40,857	286,622	0,122	291,879	30,063	18hpi	0,8273	0,8273
1dpi	3,886	0,426	2,864	1,994	6,156	0,434	7,735	3,749	1dpi	0,2752	0,8273
3dpi	1,227	0,116	1,114	94,803	386,050	0,046	370,670	108,492	3dpi	0,8273	0,8273
5dpi	1,892	0,360	1,524	0,152	1,083	0,025	2,794	1,298	5dpi	0,8273	0,1797
7dpi	1,905	0,322	1,577	1,321	11,031	0,084	12,705	4,888	7dpi	0,8273	0,4795
nimrod-like gene or putative Nimrod									nimrod		
4hpi					2,357	0,576	1,672	0,399	4hpi		0,2752
8hpi					0,950	0,176	0,895	0,118	8hpi		0,8273
18hpi					0,956	0,389	3,750	0,313	18hpi		0,0495
1dpi					1,671	0,326	0,585	0,095	1dpi		0,0495
3dpi					2,138	0,435	0,670	0,245	3dpi		0,0495
5dpi					1,038	0,108	1,391	0,080	5dpi		0,1266
7dpi					0,347	0,046	0,308	0,067	7dpi		0,8273
